# Polymorphisms of an Innate Immune Gene, Toll-Like Receptor 4, and Aggressive Prostate Cancer Risk: A Systematic Review and Meta-Analysis

**DOI:** 10.1371/journal.pone.0110569

**Published:** 2014-10-31

**Authors:** Pei-Hsuan Weng, Yi-Ling Huang, John H. Page, Jen-Hau Chen, Jianfeng Xu, Stella Koutros, Sonja Berndt, Stephen Chanock, Meredith Yeager, John S. Witte, Rosalind A. Eeles, Douglas F. Easton, David E. Neal, Jenny Donovan, Freddie C. Hamdy, Kenneth R. Muir, Graham Giles, Gianluca Severi, Jeffrey R. Smith, Carmela R. Balistreri, Irene M. Shui, Yen-Ching Chen

**Affiliations:** 1 Department of Family Medicine, Taiwan Adventist Hospital, Taipei, Taiwan; 2 Institute of Epidemiology and Preventive Medicine, College of Public Health, National Taiwan University, Taipei, Taiwan; 3 Jean Mayer US Department of Agriculture Human Nutrition Research Center on Aging, Tufts University, Boston, Massachusetts, United States of America; 4 Channing Laboratory, Department of Epidemiology, Brigham and Women's Hospital, Boston, Massachusetts, United States of America; 5 Department of Geriatrics and Gerontology, National Taiwan University Hospital, Taipei, Taiwan; 6 Center for Human Genomics, Wake Forest University School of Medicine, Winston-Salem, North Carolina, United States of America; 7 Division of Cancer Epidemiology and Genetics, National Cancer Institute, NIH, Bethesda, Maryland, United States of America; 8 Core Genotyping Facility, SAIC-Frederick, Inc., NCI-Frederick, Frederick, Maryland, United States of America; 9 Division of Cancer Epidemiology and Genetics, NCI, NIH, DHHS, Bethesda, Maryland, United States of America; 10 Department of Epidemiology and Biostatistics and Center of Human Genetics, University of California San Francisco, San Francisco, California, United States of America; 11 The Institute of Cancer Research, Sutton, United Kingdom; 12 Centre for Cancer Epidemiology, Departments of Public Health and Primary Care and Oncology, University of Cambridge, Strangeways Laboratory, Cambridge, United Kingdom; 13 Surgical Oncology (Uro-Oncology: S4), Departments of Oncology and Surgery, University of Cambridge, Addenbrooke's Hospital, Cambridge, United Kingdom; 14 Department of Social Medicine, University of Bristol, Bristol, United Kingdom; 15 Academic Urology Unit, University of Sheffield, Sheffield, United Kingdom; 16 University of Nottingham Medical School, Queens Medical Centre, Nottingham, United Kingdom; 17 Cancer Epidemiology Centre, Cancer Council Victoria, Melbourne, Australia; 18 Department of Medicine, Vanderbilt University School of Medicine, Nashville, Tennessee, United States of America; 19 Department of Pathobiology and Medical and Forensic Biotechnologies, University of Palermo, Palermo, Italy; 20 Department of Epidemiology, Harvard School of Public Health, Boston, Massachusetts, United States of America; 21 Research Center for Genes, Environment and Human Health, College of Public Health, National Taiwan University, Taipei, Taiwan; 22 Department of Public Health, College of Public Health, National Taiwan University, Taipei, Taiwan; Yale School of Public Health, United States of America

## Abstract

**Background:**

Toll-like receptor 4 (TLR4) is one of the best known TLR members expressed on the surface of several leukocytes and tissue cells and has a key function in detecting pathogen and danger-associated molecular patterns. The role of TLR4 in the pathophysiology of several age-related diseases is also well recognized, such as prostate cancer (PCa). *TLR4* polymorphisms have been related to PCa risk, but the relationship between *TLR4* genotypes and aggressive PCa risk has not been evaluated by any systematic reviews.

**Methods:**

We performed a systematic review and meta-analysis of candidate-gene and genome-wide association studies analyzing this relationship and included only white population. Considering appropriate criteria, only nine studies were analyzed in the meta-analysis, including 3,937 aggressive PCa and 7,382 controls.

**Results:**

Using random effects model, no significant association was found in the ten *TLR4* SNPs reported by at least four included studies under any inheritance model (rs2737191, rs1927914, rs10759932, rs1927911, rs11536879, rs2149356, rs4986790, rs11536889, rs7873784, and rs1554973). Pooled estimates from another ten *TLR4* SNPs reported by three studies also showed no significant association (rs10759930, rs10116253, rs11536869, rs5030717, rs4986791, rs11536897, rs1927906, rs913930, rs1927905, and rs7045953). Meta-regression revealed that study type was not a significant source of between-study heterogeneity.

**Conclusions:**

*TLR4* polymorphisms were not significantly associated with the risk of aggressive PCa.

## Introduction

Prostate cancer (PCa) is the most common malignancy since 1984, the most frequently diagnosed cancer, and the second leading cause of cancer-related deaths in 2013 among men in the USA [1]. The risk of PCa is related to family history, race, and genetic factors. Several other causes have been associated with PCa pathogenesis, including infectious agents, chronic non-infectious inflammatory diseases, diet, environmental carcinogens, imbalance of sex hormone, obesity, and urine reflux [2]–[4]. Chronic inflammation has been linked to the pathogenesis of PCa in both epidemiologic studies and molecular pathology investigations [5], [6]. In particular, several studies have suggested that sexually transmitted infections may be a risk factor for PCa through causing inflammation, even though not all the studies are consistent [7], [8]. Chronic inflammation seems to induce prostate carcinogenesis and also promote neoplastic progression [9]. Furthermore, several pathways linking inflammation and PCa have been identified: an intrinsic one driven by genetic events that cause neoplasia, and an extrinsic one driven by inflammatory conditions that predispose to cancer [9]. Among these, the eicosanoid pathway activated by cyclooxygenase 2 (COX-2) has been suggested to be involved in the pathogenesis of aggressive PCa by a recent study [10]. COX-2 was over-expressed in PCa tumors and the intensity of immunostaining was correlated with prostate tumor grade [11]. Despite the available evidence on the role of the inflammatory response in PCa onset and progression, the association between genetic variants of innate immune genes and the risk of aggressive PCa remains unclear.

Toll-like receptor 4 (TLR4) is an important pathogen recognition receptor involved in detection of lipopolysaccharide (LPS) of Gram-negative bacteria and other exogenous or endogenous ligands [12]. The TLR4 encoding gene is located on chromosome 9q32-q33. Through nuclear factor kappa B (NF-κB), TLR4 initiates the production of pro-inflammatory cytokines, such as interleukin (IL)-1, IL-6 and tumor necrosis factor-α (TNF-α) [13]. TLR4 also mediates signaling related to tumor cell invasion, survival, and metastasis in various cancers [14], [15]. Its activity and function seems to be modulated by genetic variations, principally single nucleotide polymorphisms (SNPs). Mice with deficiency or mutation of TLR4 had a weaker inflammatory immune response to viral, bacterial [16], [17], and protozoal [18] infections than that of wild-type mice. Therefore, variations in *TLR4* gene may modify the signaling of the immune response, which in turn may have effects on the pathogenesis of PCa.

Three recent meta-analyses have explored the association between *TLR4* SNPs and PCa [19]–[21]. They all reported non-significant findings after stratification by ethnicity. However, these studies focused their attention on overall PCa and did not contain genome-wide association studies (GWASs). In addition, they did not analyze the association between *TLR4* SNPs and the aggressive type of PCa. Thus, we conducted a systematic review and meta-analysis of all genetic epidemiologic association studies that have evaluated the relationship between *TLR4* polymorphisms and risk of aggressive PCa. Both candidate-gene studies and GWASs were included. The primary research questions are: (1) is there an association between *TLR4* SNPs and risk of aggressive PCa and if so, what is the size of the relationship? (2) what is the validity of the evidence of association between *TLR4* polymorphisms and risk of aggressive PCa?

## Materials and Methods

### Ethics Statement

The execution of each individual study was previously approved by the respective institution. This systematic review was performed at the study level without access to individual-level data, and therefore, institutional review board approval was not necessary. Informed consent was obtained from each participant before the start of each individual study.

### Study Selection

The study was performed using pre-specified research objectives, search strategy, study eligibility criteria, methods of data extraction, and statistical analyses. Relevant studies were identified by searching the MEDLINE (http://gateway.ovid.com/), EMBASE (http://www.embase.com), Science Citation Index (http://science.thomsonreuters.com/cgi-bin/jrnlst/jlsearch.cgi?PC=K), and Online Mendelian Inheritance in Man (http://www.ncbi.nlm.nih.gov/omim) databases for all genetic association studies published before February 2013, using combinations of the search terms “toll-like receptor 4,” OR “toll-like receptor 4 gene,” OR “*TLR*,” OR “*TLR* gene,” OR “*TLR4*,” OR “*TLR4* gene,” AND “prostate cancer,” OR “prostatic neoplasms.” GWASs were searched using combinations of the search terms “genome-wide association study,” OR “GWAS,” AND “prostate cancer,” OR “prostatic neoplasms.” In addition, we manually searched the reference lists from reviews and original articles to retrieve other papers relevant to the topic. Where there was overlap in the study populations of published papers, only the largest study was included. No language restriction was placed on the literature search strategies. Unpublished findings were not identified.

### Exposure Measures

The main exposure variables were *TLR4* genotypes as measured in blood DNA samples from men in the respective studies. This meta-analysis summarized *TLR4* SNPs which were reported by at least three included studies. Because many *TLR4* SNPs were explored by two studies only, and the respective sample sizes were small, these SNPs were not analyzed in this meta-analysis.

### Outcome Measures

The outcome measure was aggressive PCa as defined by Gleason score greater than or equal to seven, or TNM stage greater than or equal to T3b or any nodal involvement or any distant metastases. However, some included studies extended this definition. Controls for aggressive PCa are ideally men without aggressive PCa chosen from the population at risk, although some studies selected controls from men without screening for occult PCa (Table 1).

**Table 1:** Characteristics of the study populations that evaluated the relationship between *TLR4* polymorphisms and risk of prostate cancer.

Source, publication year (study year)	Type of study	Country/ancestry	Aggressive PCa/control	Control selection	Comments about control selection	Case selection	Definition of aggressive prostate cancer	Outcome assessment “blinded” to genotype	Genotyping procedures	Genotyping quality control
Chen et al., 2005 (1993–1995)	Candidate gene	U.S./97% Caucasians	260/700	Age- matched controls from prospective cohort	PSA tested in controls	Incident PCa	TNM stage T3b or T4 or N1 or M1 or death due to PCa or Gleason sum ≥7	Yes	MassARRAY system (SEQUENOM)**	100% concordance,>95% genotyping success
Dunggan et al., 2007 (2001–2002)	GWAS	Sweden/Not mentioned	505/507	Age-matched population controls from the same geographical region	74% response rate in cases, 52% in controls. No PSA tested in controls.	PCa from cancer registry	TNM stage T3 or T4 or N+ or M+ or grade III or Gleason sum>7 or PSA>100 ng/ml	Yes	MassARRAY system (SEQUENOM)**	>99% concordance,>98% genotyping success
Yeager et al., 2007 (1993–2001)	GWAS	U.S./White and non-hispanic	1081/1416	Risk set sampling from a population-based randomized controlled trial	PSA tested in controls	Incident PCa	Gleason sum ≥7 or stage ≥3	Yes	Illumina system	>99% concordance,>99% genotyping success
Cheng et al., 2007 (2002–2004)	Candidate gene	U.S./Caucasians	417/417	From annual medical examinations at the same medical institutions of cases	Hospital-based study. PSA tested in controls	Incident PCa	TNM stage ≥ T2c or Gleason sum ≥7 or PSA>10 ng/ml	Yes	Taqman	100% concordance, 99.9% genotyping success
Eeles et al., 2008 (1993–2001)	GWAS	U.K., Australia/Excluded self-reported “non-white”	564/1894	Community-based randomized controlled trial/electoral rolls	Controls to be frequency matched to the geographical distribution of the cases.	PCa from cancer registry, urology clinic	Gleason sum ≥7	Yes	Stage 1: Illumina Infinium HumanHap550 array. Stage 2: Taqman	>97% SNPs at a confidence score of ≥0.25, 98.8% concordance
Breyer et al.,2009 (2002–2008)	Candidate- gene	U.S./Americans of Northern European decents	441/772	Age-matched controls from a preventive screening	Hospital-based. PSA tested in controls	Incident PCa	Gleason sum ≥7	Yes	Illumina GoldenGate platform and Taqman	99.7% of genotyping success
Wang et al., 2009 (1992–2002)	Candidate gene	U.S./White only	77/264	Age- matched controls from a prospective cohort	No PSA tested in controls	Incident PCa	TNM stage T3 or T4 or N1 or M1 or death due to PCa or Gleason sum ≥7	Not mentioned	Taqman	93–99% genotyping success
Ballistreri et al., 2010 (NA)	Candidate gene	Italy/European ancestry	32/125	Age-matched controls in good health	Hospital-based study. No clear description on control selection. No PSA tested in controls	Prevalent PCa	Gleason sum ≥7	Yes	RFLP-PCR	Not mentioned
Shui et al., 2012 (1982–2004)	Candidate gene	U.S./White	560/1287	Risk set sampling from a prospective cohort, matched on age and smoking	No PSA tested in controls	Incident PCa	TNM stage T3 or T4, M1 or N1 or death due to PCa or Gleason sum ≥7	Yes	Sequenom iPLEX matrix-assisted laser desorption/ionization time of flight (MALDI-TOF) mass spectrometry technology.	100% concordance,>95% genotyping success

Abbreviations: PCa, prostate cancer; TNM, the tumor node metastases classification system; PSA, prostate specific antigen; GWAS, genome-wide association study; RLFP-PCR, restriction fragment length polymorphism-polymerase chain reaction.

All studies met the following criteria and they were not listed in the table: (1) clear description of laboratory methods, (2) genotyping identical for cases and controls, (3) genotyping blinded to case control status, and (4) specimen came from peripheral blood sample.

### Data Extraction

Three of us (PH Weng, YL Huang, and YC Chen) independently reviewed each published paper and extracted relevant information examining the associations between *TLR4* polymorphisms and risk of aggressive PCa. Inter-observer differences, if any existed, were reconciled through group discussion. In order to pool data from different studies, we requested data from each study based on the definition for aggressive PCa in this meta-analysis, which may be slightly different from their original design. For GWASs that did not report detailed information of *TLR4*, we contacted the investigators to obtain data on advanced PCa counts and the corresponding *TLR4* genotyping frequencies. To avoid population stratification, this meta-analysis was restricted to samples taken from European ancestry. We evaluated selection bias based on the extent to which controls are representative of the “person-time population” from which the cases were sampled, and the extent to which cases are a random sample of that latter population.

### Statistical Analyses

Meta-analyses were performed for SNPs that were reported by at least three included studies. The pooled odds ratios (ORs) and 95% confidence intervals (CIs) for the associations between *TLR4* genotypes and risk of aggressive PCa were calculated using random effects models. Random effects models are preferred to fixed effect models because of the differences in study designs and study populations [22]. To incorporate both within-study and between-study variability, we used DerSimonian and Laird's [23] random effects models to pool the estimates of log OR from each individual study (unadjusted for covariates). Between-study heterogeneity was quantified by using the I^2^ statistic [24], [25], which indicates the proportion of variability across studies attributable to heterogeneity. Tests of heterogeneity were assessed by a χ^2^ statistic. To explore the inheritance mode for the effect of *TLR4* polymorphisms, we evaluated the following genotype contrasts (where a and A denote minor and major alleles, respectively): (1) a/a and A/a combined versus A/A (dominant model); (2) a/a versus A/a and A/A combined (recessive model); (3) a/a versus A/A and A/a versus A/A (co-dominant model); (4) the increment of one minor allele (additive model). The Hardy-Weinberg equilibrium (HWE) was assessed via χ^2^ test. We did not perform haplotype analysis because none of the previous studies performed haplotype analysis specific for these SNPs. Because most GWASs did not adjust for covariates, this meta-analysis reported unadjusted pooled results.

To evaluate the presence of publication bias, we examined the funnel plot, by plotting the reciprocal of the standard error of log OR versus the log OR, for symmetry. The Egger linear regression test was also performed to assess funnel plot's asymmetry [26]. Random effects meta-regression was performed under dominant model to explore possible sources of between-study heterogeneity. Study type (candidate-gene studies vs. GWASs) was the pre-specified covariate. We did not perform stratification analysis according to differences in control and case selection, because such influences are complex and are usually not unidirectional. Because previous studies revealed high concordance rate across genotyping platforms [27], stratification analysis was not carried out according to this covariate. Analyses were performed with Stata version 11.0 software (Stata Corporation, College Station, TX, USA). All *P* values were two-sided. QUANTO program (http://hydra.usc.edu/gxe/) was used to evaluate statistical power of the association between *TLR4* polymorphisms and aggressive PCa.

## Results

### Characteristics of Association Studies

**Figure 1:**
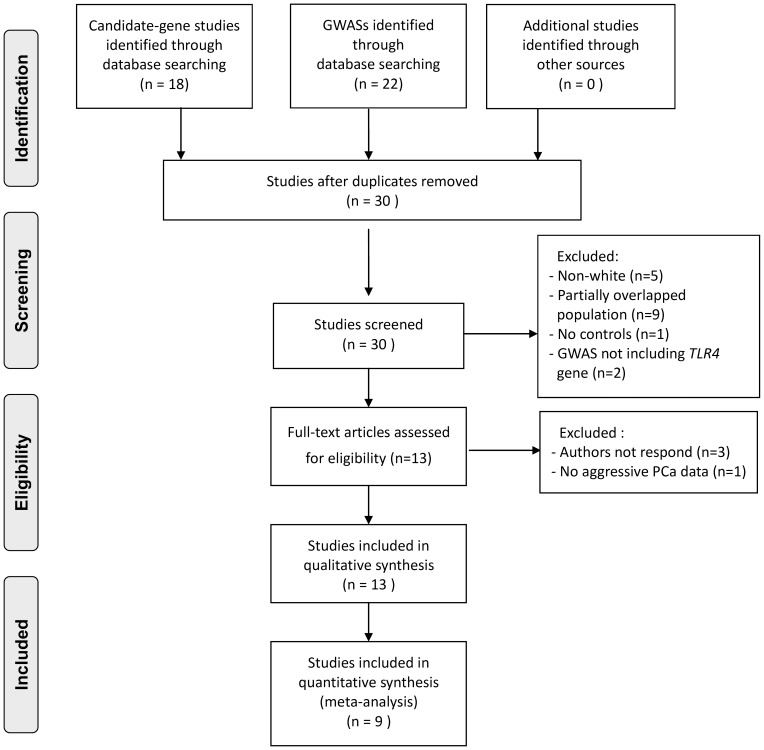
Study selection flowchart. Forty studies were reviewed after literature search. Among them, 31 studies were excluded due to duplication, race other than whites, and insufficient data. A total of 9 studies were included for meta-analysis.

Using the pre-specified search methodology we retrieved forty relevant publications (Figure 1). After excluding duplicates (n = 10), seventeen studies were further excluded due to the following reasons: (1) not European ancestry (n = 5), (2) partially overlapped populations (n = 9), (3) lack of controls (n = 1), and (4) GWAS which did not include *TLR4* gene (n = 2).

We contacted the authors of the remaining 13 relevant studies for necessary details, and authors of three of the GWASs [28]–[30] didn't respond and were thus excluded. One GWAS was excluded because it didn't contain the information of PCa aggressiveness [31]. For studies composed of multiple cohorts (e.g., Lindstrom et al. [32]), we tried to obtain data from each cohort and used the original study to represent each cohort (e. g., Chen et al. [33] for HPFS, Dunggan et al. [34] for CAPS, and Yeager et al. [35] for PLCO). For the CAPS study, the GWAS by Dunggan et al. [34] was selected instead of the candidate-gene study done by Zheng et al. [36] because the former was composed of aggressive PCa cases from Zheng's study and evaluated more SNPs. In sum, nine studies were included for the meta-analysis.

A total of 3,937 aggressive PCa cases and 7,382 controls were included in this work. Six studies were candidate-gene studies [33], [37]–[41], and three of them were GWASs [34], [35], [42]. Six papers studied US populations [33], [35], [37]–[39], one studied a Swedish population [34], one studied the combination of UK and Australian population [42], and one studied an Italian population [40]. Details of the studies analyzed in this meta-analysis were summarized in Table 1, including first author, year of publication, type of study, ancestry, sample size, control selection, possible sources of selection bias, definition of PCa aggressiveness, genotyping methods and quality control.

For the association between *TLR4* SNPs and aggressive PCa, seven studies assessed rs4986790 [33]–[35], [37], [39], [40], [42]; five studies investigated rs2149356 [33], [34], [37], [39], [41], rs11536889 [33], [34], [37], [39], [41], rs7873784 [33], [34], [37], [39], [41]; and four studies explored rs2737191[34], [35], [41], [42], rs1927914 [33], [34], [38], [39], rs10759932 [33], [34], [37], [41], rs1927911 [33], [34], [38], [39], rs11536879 [34], [35], [38], [42], and rs1554973 [34], [35], [41], [42].

### Allele Frequencies of *TLR4* SNPs

**Figure 2:**
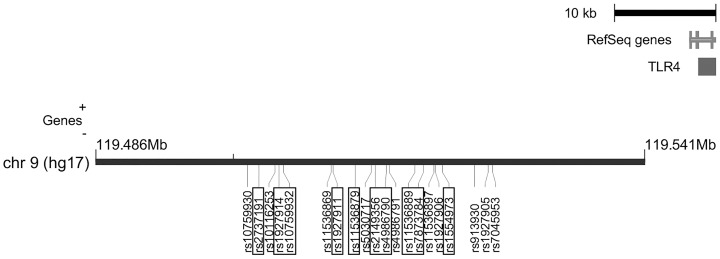
*TLR4* SNPs evaluated in this meta-analysis. This plot was generated by the Locusview program. The highlighted boxed SNPs were *TLR4* polymorphisms explored by at least four studies. The remaining SNPs were those reported by three studies, discussed in the supplemental data.

Ten *TLR4* SNPs had been evaluated by at least 4 included studies. The minor allele frequencies (MAF) between case and controls were shown in Table 2, along with the test for HWE in controls. Among them, three SNPs are located on 5′ untranslated region (UTR, rs2737191, rs1927914 and rs10759932), three are intronic SNPs (rs1927911, rs11536879, and rs2149356), one is non-synonymous exonic SNP (rs4986790), and three SNPs are located on 3′ UTR (rs11536889, rs7873784, and rs1554973). Another 10 *TLR4* SNPs were reported by 3 studies, including one SNP located on the promoter region (rs10759930), one SNP located on 5′UTR (rs10116253), two intronic SNPs (rs11536869 and rs5030717), one non-synonymous exonic SNP (rs4986791), and five SNPs located on 3′ UTR (rs11536897, rs1927906, rs913930, rs1927905, and rs7045953). The locations of the explored SNPs (10 SNPs with ≥4 studies, 10 SNPs with 3 studies) are shown in Figure 2. rs2149356, rs4986790 and rs7873784 in Chen's study and rs1927911 in Wang's study were out of HWE (*P* = 0.01–0.03) but were kept in the analysis because the HWE tests were not significant after correction for multiple tests.

**Table 2:** Characteristics of included studies.

	rs2737191 (A/G)		rs1927914 (A/G)		rs10759932 (T/C)		rs1927911 (G/A)		rs11536879 (A/G)		rs2149356 (G/T)		rs4986790 (A/G)		rs11536889 (A/G)		rs7873784 (G/C)		rs1554973 (T/C)	
	MAF case/control	HWE *P* in controls	MAF case/control	HWE *P* in controls	MAF case/control	HWE *P* in controls	MAF case/control	HWE *P* in controls	MAF case/control	HWE *P* in controls	MAF case/control	HWE *P* in controls	MAF case/control	HWE *P* in controls	MAF case/control	HWE *P* in controls	MAF case/control	HWE *P* in controls	MAF case/control	HWE *P* in controls
Chen et al.,2005	NA	NA	0.30/0.35	0.15	0.14/0.16	0.09	0.25/0.29	0.43	NA	NA	0.30/0.34	0.02	0.04/0.05	0.01	0.15/0.14	0.52	0.15/0.18	0.03	NA	NA
Dunggan et al.,2007	0.27/0.27	0.46	0.33/0.34	0.55	0.16/0.15	0.71	0.27/0.26	0.74	0.01/0.01	0.82	0.31/0.32	0.89	0.05/0.06	0.15	NA	NA	0.11/0.13	0.99	0.19/0.21	0.45
Yeager et al.,2007	0.28/0.29	0.88	0.32/0.32	0.94	NA	NA	NA	NA	0.04/0.04	0.83	NA	NA	0.06/0.05	0.59	NA	NA	NA	NA	0.24/0.23	0.11
Cheng et al.,2007	NA	NA	NA	NA	0.13/0.14	0.04	NA	NA	NA	NA	0.32/0.30	0.68	0.06/0.05	0.98	0.15/0.14	0.09	0.15/0.16	0.82	NA	NA
Eeles et al.,2008	0.27/0.29	0.76	0.33/0.33	0.79	NA	NA	NA	NA	0.05/0.04	0.71	NA	NA	0.05/0.06	0.74	NA	NA	NA	NA	0.26/0.26	0.86
Breyer et al, 2009	NA	NA	NA	NA	NA	NA	0.27/0.26	0.34	0.04/0.03	0.92	NA	NA	NA	NA	NA	NA	NA	NA	NA	NA
Wang et al.,2009	NA	NA	0.32/0.32	0.24	NA	NA	0.27/0.24	0.02	NA	NA	0.35/0.32	0.18	0.06/0.07	0.24	0.16/0.16	0.76	0.11/0.12	0.91	NA	NA
Ballistreri et al.,2010	NA	NA	NA	NA	NA	NA	NA	NA	NA	NA	NA	NA	0/0.06	0.38	NA	NA	NA	NA	NA	NA
Shui et al.,2012	0.26/0.26	0.08	NA	NA	0.13/0.13	0.03	NA	NA	NA	NA	0.30/0.30	0.06	NA	NA	0.16/0.14	0.55	0.14/0.14	0.20	0.25/0.25	0.18

SNPs that were evaluated by at least 4 studies were shown here. Abbreviations: MAF, minor allele frequency; HWE, Hardy–Weinberg

equilibrium; NA, not available.

### Meta-Analysis

**Figure 3:**
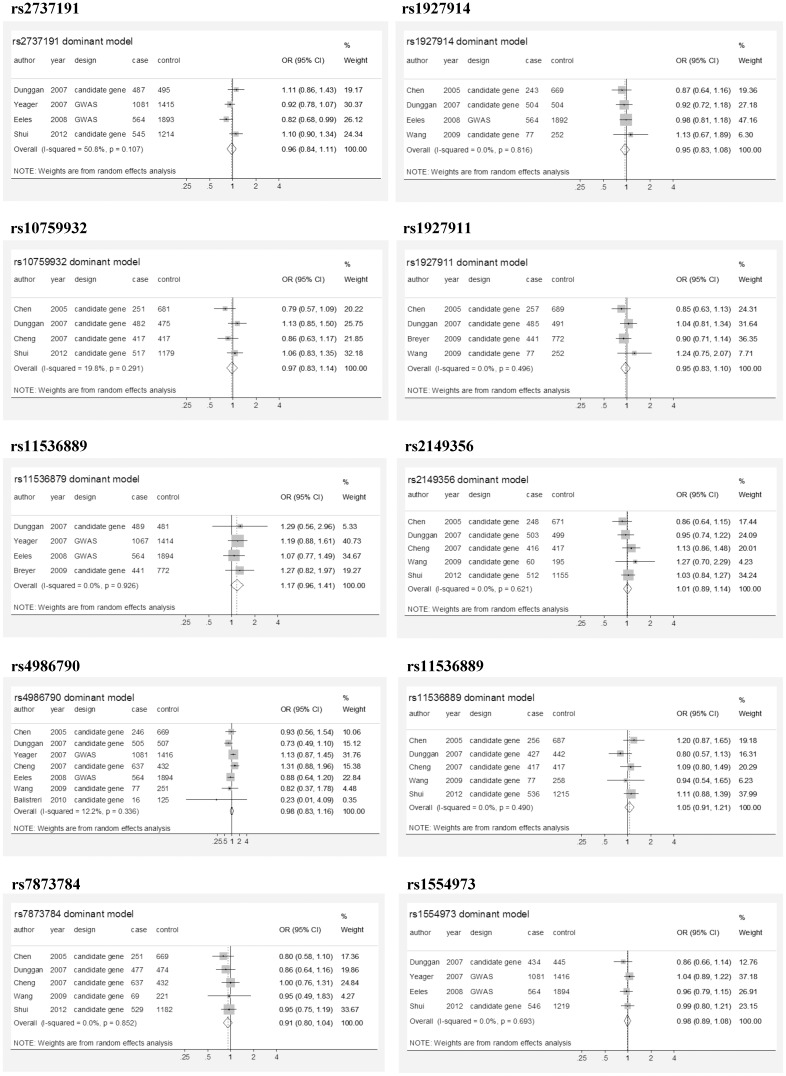
Forest plot examines relationship between *TLR4* SNPs and risk of aggressive prostate cancer. Odds ratios and weights were demonstrated for each individual study and for the pooled analysis, assuming a dominant model. SNPs that were evaluated by at least 4 studies were shown here.

This meta-analysis was reported according to the PRISMA checklist [43] (Checklist S1). Using random effects meta-analysis, the ten *TLR4* SNPs (rs2737191, rs1927914, rs10759932, rs1927911, rs11536879, rs2149356, rs4986790, rs11536889, rs7873784, and rs1444973) were not associated with the risk of aggressive PCa regardless of the inheritance model used (Table 3, Figure 3). The meta-analysis was also performed for another ten SNPs which were reported by three included studies (rs10759930, rs10116253, rs11536869, rs5030717, rs4986791, rs11536897, rs1927906, rs913930, rs1927905, and rs7045953) (Table S1). None of the SNPs revealed significant association with aggressive PCa.

**Table 3:** Pooled estimated ORs and 95% CIs for the association of *TLR4* SNPs in aggressive PCa risk.

		Random effects model		Heterogeneity				Random effects model		Heterogeneity	
	Genetic model	OR (95% CI)	*P*	I^2^	*P*		Genetic model	OR (95% CI)	*P*	I^2^	*P*
rs2737191	Dominant	0.96 (0.84–1.11)	0.61	50.8%	0.11	rs2149356	Dominant	1.01 (0.89–1.14)	0.90	0%	0.62
	Recessive	0.86 (0.72–1.03)	0.40	0%	0.40		Recessive	0.91 (0.73–1.12)	0.37	6%	0.37
	AG vs. AA	1.00 (0.83–1.19)	0.97	65.9%	0.03		GT vs. GG	1.03 (0.91–1.17)	0.63	0%	0.86
	GG vs. AA	0.84 (0.84–1.08)	0.07	35.7%	0.14		TT vs. GG	0.92 (0.72–1.17)	0.49	16.9%	0.31
	Additive	0.95 (0.84–1.07)	0.41	30%	0.18		Additive	0.99 (0.90–1.08)	0.83	0%	0.69
rs1927914	Dominant	0.95 (0.83–1.08)	0.43	0%	0.82	rs4986790	Dominant	0.98 (0.83–1.16)	0.82	12.2%	0.34
	Recessive	0.88 (0.62–1.24)	0.46	52.9%	0.10		Recessive	1.29 (0.57–2.95)	0.55	0%	0.81
	AG vs. AA	0.96 (0.84–1.10)	0.53	0	0.86		AG vs. AA	0.98 (0.83–1.16)	0.81	10%	0.35
	GG vs. AA	0.87 (0.63–1.21)	0.41	44.9%	0.14		GG vs. AA	1.28 (0.56–2.93)	0.59	0%	0.82
	Additive	0.96 (0.87–1.06)	0.44	1%	0.42		Additive	1.02 (0.88–1.17)	0.83	0%	0.62
rs10759932	Dominant	0.97 (0.83–1.14)	0.70	19.8%	0.29	rs11536889	Dominant	1.05 (0.91–1.21)	0.48	0%	0.49
	Recessive	1.33 (0.70–2.54)	0.38	44%	0.15		Recessive	1.25 (0.84–1.86)	0.26	0%	0.94
	TC vs. TT	0.94 (0.79–1.14)	0.54	35.7%	0.20		AG vs. AA	1.03 (0.89–1.20)	0.66	0%	0.48
	CC vs. TT	1.31 (0.70–2.46)	0.40	40.9%	0.17		GG vs. AA	1.27 (0.85–1.89)	0.24	0%	0.95
	Additive	0.96 (0.81–1.13)	0.60	20.5%	0.27		Additive	1.06 (0.94–1.20)	0.32	0%	0.87
rs1927911	Dominant	0.95 (0.83–1.10)	0.49	0%	0.50	rs7873784	Dominant	0.91 (0.80–1.05)	0.19	0%	0.85
	Recessive	1.06 (0.67–1.67)	0.80	56.2%	0.08		Recessive	1.03 (0.69–1.52)	0.90	0%	0.56
	GA vs. GG	0.93 (0.80–1.08)	0.35	0%	0.44		GC vs. GG	0.91 (0.79–1.04)	0.17	0%	0.88
	AA vs. GG	1.03 (0.67–1.61)	0.88	51.1%	0.11		CC vs. GG	1.00 (0.67–1.48)	0.99	0%	0.55
	Additive	0.99 (0.84–1.17)	0.92	23.9%	0.24		Additive	0.93 (0.83–1.05)	0.26	0%	0.84
rs11536879	Dominant	1.17 (0.96–1.41)	0.12	0%	0.93	rs1554973	Dominant	0.98 (0.89–1.08)	0.71	0%	0.69
	Recessive	0.82 (0.17–3.86)	0.80	0%	0.80		Recessive	1.01 (0.83–1.24)	0.91	0%	0.86
	AG vs. AA	1.18 (0.97–1.43)	0.10	0%	0.95		TC vs. TT	0.98 (0.88–1.08)	0.67	0%	0.75
	GG vs. AA	0.83 (0.18–3.91)	0.82	0%	0.45		CC vs. TT	1.01 (0.82–1.23)	0.96	0%	0.83
	Additive	1.15 (0.95–1.40)	0.15	0%	0.95		Additive	0.99 (0.92–1.07)	0.81	0%	0.95

SNPs that were evaluated by at least 4 studies were shown here. Abbreviation: OR, odds ratio; CI, confidence interval; PCa, prostate cancer.

### Publication Bias

Funnel plots were used to assess the relationship between the ten *TLR4* SNPs and aggressive PCa (Figure S1). Using the Egger linear regression test, possible publication bias was found among the included studies on rs1554973 (Egger test *P* = 0.06). For the other 9 SNPs, *P* values ranged from 0.2 to 0.77.

### Meta-Regression

Random effects meta-regression was performed under dominant model. Different study type (candidate-gene studies vs. GWASs) was not a significant source of between-study heterogeneity (*P* value ranged from 0.15 to 0.79 for the ten *TLR4* SNPs).

### Power Calculation

For people of European ancestry, given a MAF of 0.15 and α of 0.05, this study had over 95% power to detect an OR of 1.20 for 3,937 cases and 7,382 controls.

## Discussion

Recently, some researchers hypothesized that PCa is the result of a chronic inflammatory process [44]. Proliferative inflammatory atrophy (PIA), proposed as a potential precursor to PCa, occurs frequently in the periphery of the prostate gland where PCa occurs [5]. PIA lesions seem to be the result of different conditions, including infections, chronic non-infectious inflammatory diseases, dietary carcinogens, physical trauma, imbalance of sex hormone and urine reflux [9]. Chronic infections may contribute to PIA and lead to onset of PCa [45]–[47]. Several innate inflammatory pathways seem to be involved. Among these, TLR4 pathway plays a crucial role [48].

**Figure 4:**
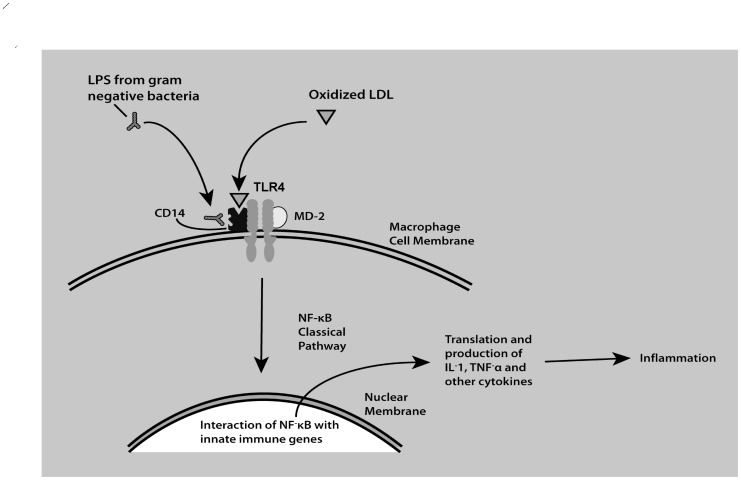
The role of TLR4 in innate immunity. TLR4 receptors are responsible for the recognition of bacterial lipopolysaccharide (LPS) monomers and partially oxidized LDL (oLDL) on innate immune cells. LPS monomers and oLDL bind to sites on the protein, CD14. CD14 promotes the binding of these ligands to the TLR4-MD-2 complex, which signals the activation of the nuclear factor kappa B (NF-κB) pathway. NF-κB products enter the nucleus and result in transcription followed by the production of cytokines and the activation of multiple inflammatory pathways. This figure was adapted from DeFranco et al. [48].

TLR4 recognizes pathogen-associated molecular patterns, i.e. LPS [46]. Damage-associated molecular pattern molecules may also interact with TLR4, i.e. oxidized low-density lipoprotein (LDL) [49], one of the atherogenic lipoproteins associated with atherosclerosis [50] and insulin resistance [51], [52]. Their interaction leads to the initiation of inflammatory response via NF-κB (Figure 4) [53]. TLR4 can also promote PCa development through releasing inflammatory mediators. Associations between *TLR4* SNPs and PCa have been examined in several studies, though discordant data have been reported. However, the relationship between *TLR4* genotypes and aggressive PCa risk has not been evaluated by any systematic reviews. Thus, we performed a systematic review and meta-analysis of candidate-gene studies and GWASs analyzing this relationship and restricted to samples taken from European ancestry.

In the current meta-analysis, none of the examined *TLR4* SNPs was significantly associated with risk of aggressive PCa under any inheritance model. No significant association was found between the *TLR4* SNPs (5′UTR: rs2737191, rs1927914 and rs10759932; intron: rs1927911, rs11536879, and rs2149356; exon: rs4986790; 3′UTR: rs11536889, rs7873784, and rs1554973) and risk of aggressive PCa in the pooled analysis. The non-significant findings may be attributable to (1) failure to adjust for the conventional risk factors of PCa, e.g. family history of PCa, (2) inability to assess the within-population heterogeneity or geographic variation, and (3) the studied *TLR4* SNPs may be more closely related to non-aggressive PCa.

Three recent meta-analyses evaluated the association between *TLR4* SNPs and overall PCa. Jing et al. [19], including four candidate-gene studies [33], [37], [39], [40], examined two *TLR4* SNPs (rs4986790 and rs4986791) and found that rs4986790 showed a protective effect on overall PCa under co-dominant and recessive models. However, the effect was not statistically significant after stratification by ethnicity. Another work by Zhang et al. [20] examined six *TLR4* SNPs (rs1927914, rs4986790, rs4986791, rs11536889, rs1927911, rs2149356) and did not find significant associations with overall PCa. The pooled estimates of Zhang et al. were derived from one Asian study [54] and four other populations of European ancestry [33], [36], [39], [41], which might be confounded by population stratification. Zhu et al. [21] examined rs4986790 and rs4986791 and found no significant association with overall PCa in five populations of European ancestry [33], [36], [37], [39], [40]. In summary, our findings on aggressive PCa are consistent with the previous meta-analyses on overall PCa. Our study had several advantages over the previous meta-analyses: (1) this study additionally included GWASs, whereas previous meta-analyses included candidate-gene studies only [19]–[21], (2) this study focused on aggressive PCa, which is more clinically relevant, (3) this study was restricted to populations of European ancestry to avoid population stratification, and (4) this study evaluated an additional 14 SNPs, which were not reported in the previous meta-analyses.

Previous candidate-gene studies and GWASs found inconsistent results for the association between *TLR4* polymorphisms and PCa risk. This may be explained by different ethnicity, within-population heterogeneity, case and control selection, gene-gene interactions, and gene-environment interactions. Although most of the relevant medical centers were in the “catchment” area, Cheng and colleagues [37] used controls from medical centers, which differ from the source population in that not all men with potential PCa would go to these centers to be screened and diagnosed.

There were some limitations of this study. One of them is the possibility of publication bias. Though the funnel plots did not reveal obvious publication bias among most of *TLR4* SNPs, the SNPs reported in this study were under the influence of publication bias because only SNPs explored in ≥3 studies were included. We were unable to include three other GWASs because the authors did not respond to our data request [28]–[30]. After exclusion of men with African and Asian ancestry, there was little evidence that population stratification was a cause of confounding. Though the included studies were conducted separately in the United States, Sweden, Italy, UK and Australia, a prior theoretical calculation on genetic case-control studies showed that ignoring ethnicity among non-Hispanic U.S. Caucasians with ancestries from different European countries resulted in bias of less than 1% [55]. Last, the included studies used different genotyping approaches, which may be associated with different genotyping success rates and data quality. However, genotyping errors are expected to be small, and thus the resulting biases are likely to be small.

This study had some advantages. To the best of our knowledge, this is the first meta-analysis on *TLR4* polymorphisms and aggressive PCa, which shows more clinical relevance. All the included studies were reasonably well-designed epidemiological studies. Genotyping was carried out “blind” to the disease status, and assessment of aggressive PCa was carried out “blind” to the genotypes. This study had sufficient power (>0.95) to detect a potential OR of aggressive PCa associated with a SNP of 1.20. This study presents the best available evidence on the relationship between *TLR4* polymorphisms and risk of aggressive PCa.

In conclusion, this study found that none of the examined *TLR4* SNPs were significantly associated with risk of aggressive PCa under any mode of inheritance. Control selection, different ancestry, small statistical power in some studies, publication bias, gene-gene and gene-environment interactions, different genotyping approaches, and issues of multiple tests may contribute to the inconsistent findings in previous studies. Meta-regression revealed that different study type (candidate-gene studies vs. GWASs) was not a significant source of between-study heterogeneity. Large-scale and well-designed studies using population-based controls and more studies in each ethnic group are needed to confirm our findings.

## Supporting Information

Figure S1 **Funnel plot of** ***TLR4*** **SNPs.** Funnel plot displays the publication bias for each study (indicated as one dot) exploring the relation between *TLR4* SNPs and aggressive prostate cancer. SNPs reported by at least four studies were shown here.
(TIF)

Table S1 **Pooled estimated ORs and 95% CIs for the association of** ***TLR4*** **SNPs in aggressive PCa risk.**
(DOC)

Checklist S1 **PRISMA checklist.**
(DOC)
