# Supplementary figures and images for: Polymorphisms of an Innate Immune Gene, Toll-Like Receptor 4, and Aggressive Prostate Cancer Risk: A Systematic Review and Meta-Analysis
Source: PLoS One. 2014 Oct 31;9(10):e110569. doi: 10.1371/journal.pone.0110569 (PMC4215920; doi:10.1371/journal.pone.0110569)

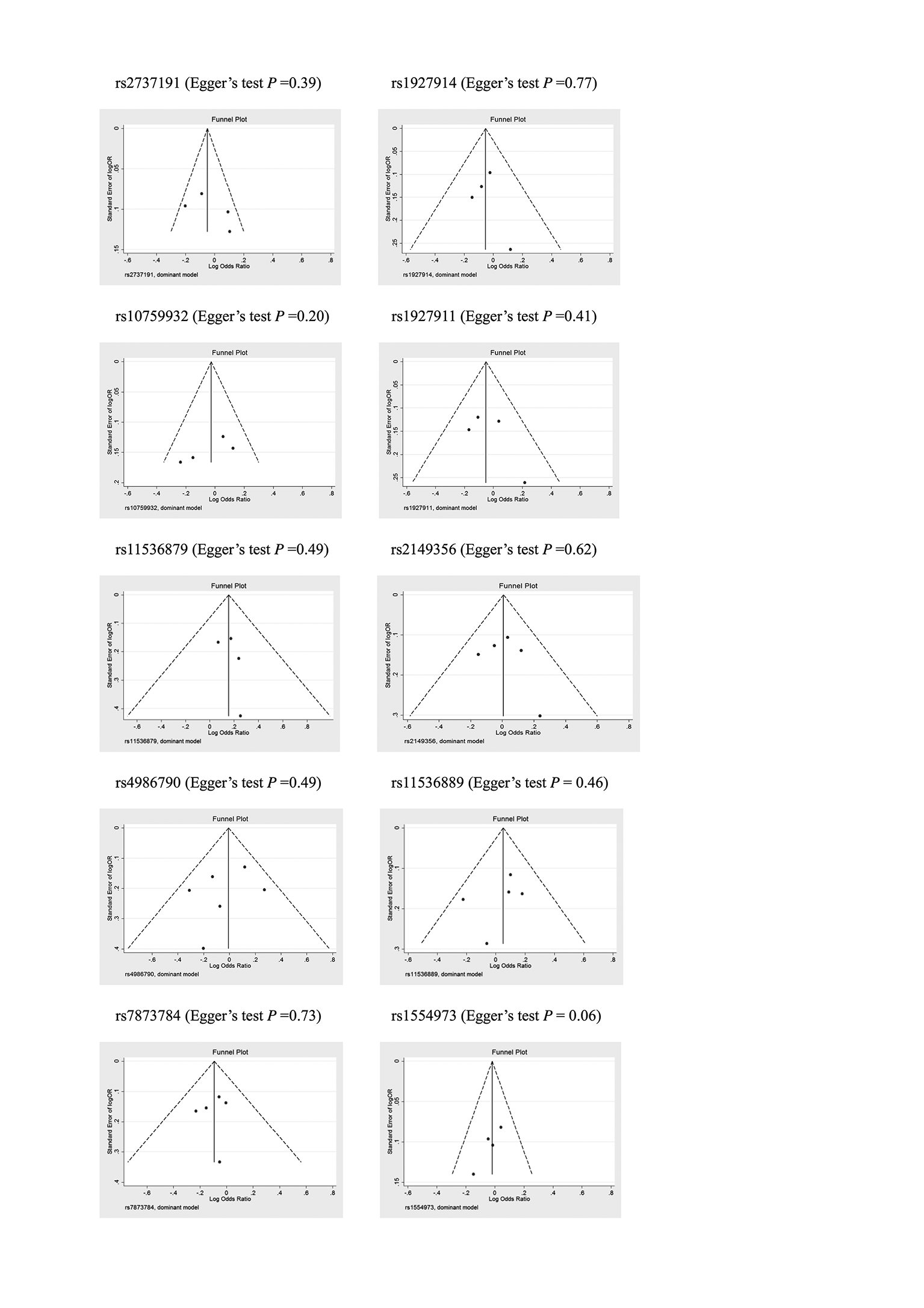

Supplement: Figure S1 — Funnel plot of TLR4 SNPs. Funnel plot displays the publication bias for each study (indicated as one dot) exploring the relation between TLR4 SNPs and aggressive prostate cancer. SNPs reported by at least four studies were shown here. (TIF) [file pone.0110569.s001.tif]
