# Supplementary material for: Polymorphisms of an Innate Immune Gene, Toll-Like Receptor 4, and Aggressive Prostate Cancer Risk: A Systematic Review and Meta-Analysis
Source: PLoS One. 2014 Oct 31;9(10):e110569. doi: 10.1371/journal.pone.0110569 (PMC4215920; doi:10.1371/journal.pone.0110569)
Supplement: Table S1 — Pooled estimated ORs and 95% CIs for the association of TLR4 SNPs in aggressive PCa risk. (DOC) [file pone.0110569.s002.doc]

**Table S1. Pooled estimated ORs and 95% CIs for the association of *TLR4* SNPs in aggressive PCa risk**

|  |  | Random effects model | |  | Heterogeneity | |  |  |  | Random effects model | |  | Heterogeneity | |
| --- | --- | --- | --- | --- | --- | --- | --- | --- | --- | --- | --- | --- | --- | --- |
|  | Genetic model | OR (95% CI) | *P* |  | I^2^ | *P* |  |  | Genetic model | OR (95% CI) | *P* |  | I^2^ | *P* |
| rs10759930 | Dominant | 1.10 (0.99-1.24) | 0.07 |  | 0% | 0.73 |  | rs11536897 | Dominant | 1.06 (0.83-1.35) | 0.63 |  | 0% | 0.60 |
|  | Recessive | 1.08 (0.93-1.25) | 0.35 |  | 0% | 0.51 |  |  | Recessive | 0.83 (0.24-2.96) | 0.78 |  | 0% | 0.89 |
|  | CT vs. CC | 1.10 (0.98-1.24) | 0.11 |  | 0% | 0.54 |  |  | GA vs. GG | 1.07 (0.84-1.37) | 0.58 |  | 0% | 0.66 |
|  | TT vs. CC | 1.14 (0.96-1.34) | 0.13 |  | 0% | 0.67 |  |  | AA vs. GG | 0.84 (0.24-.296) | 0.78 |  | 0% | 0.64 |
|  | Additive | 1.07 (0.99-1.16) | 0.08 |  | 0% | 0.72 |  |  | Additive | 1.06 (0.84-1.33) | 0.65 |  | 0% | 0.87 |
| rs10116253 | Dominant | 0.97 (0.82-1.16) | 0.77 |  | 0% | 0.47 |  | rs1927906 | Dominant | 1.03 (0.88-1.20) | 0.74 |  | 12.1% | 0.32 |
|  | Recessive | 0.84 (0.50-1.44) | 0.53 |  | 41.6% | 0.18 |  |  | Recessive | 1.19 (0.67-2.10) | 0.55 |  | 0% | 0.99 |
|  | TC vs. TT | 1.00 (0.83-1.20) | 0.99 |  | 0% | 0.74 |  |  | GA vs. AA | 1.02 (0.87-1.20) | 0.82 |  | 11.6% | 0.32 |
|  | CC vs. TT | 0.85 (0.48-1.50) | 0.57 |  | 46.1% | 0.16 |  |  | GG vs. AA | 1.20 (0.68-2.12) | 0.54 |  | 0% | 0.99 |
|  | Additive | 0.96 (0.83-1.110 | 0.60 |  | 0% | 0.47 |  |  | Additive | 1.04 (0.91-1.19) | 0.58 |  | 0% | 0.77 |
| rs11536869 | Dominant | 0.87 (0.67-1.12) | 0.28 |  | 0% | 0.56 |  | rs913930 | Dominant | 0.91 (0.76-1.10) | 0.33 |  | 59.1% | 0.09 |
|  | Recessive | 1.63 (0.38-6.93) | 0.51 |  | 0% | 0.58 |  |  | Recessive | 0.96 (0.81-1.14) | 0.64 |  | 0% | 0.73 |
|  | AG vs. AA | 0.85 (0.65-1.11) | 0.23 |  | 0% | 0.43 |  |  | TC vs. TT | 0.92 (0.73-1.16) | 0.50 |  | 70.6% | 0.03 |
|  | GG vs. AA | 1.61 (0.38-6.86) | 0.52 |  | 0% | 0.59 |  |  | CC vs. TT | 0.89 (0.75-1.07) | 0.21 |  | 0% | 0.99 |
|  | Additive | 0.89 (0.69-1.14) | 0.36 |  | 0% | 0.59 |  |  | Additive | 0.94 (0.82-1.08) | 0.37 |  | 37% | 0.16 |
| rs5030717 | Dominant | 0.94 (0.75-1.18) | 0.60 |  | 35.8% | 0.21 |  | rs1927905 | Dominant | 1.13 (0.94-1.35) | 0.21 |  | 0% | 0.94 |
|  | Recessive | 1.08 (0.50-2.34) | 0.85 |  | 0% | 0.57 |  |  | Recessive | 0.52 (0.14-1.97) | 0.33 |  | 0% | 0.91 |
|  | AG vs. AA | 0.94 (0.76-1.16) | 0.55 |  | 19.5% | 0.29 |  |  | GA vs. AA | 1.15 (0.95-1.38) | 0.15 |  | 0% | 0.93 |
|  | GG vs.AA | 1.08 (0.50-2.35) | 0.85 |  | 0% | 0.53 |  |  | GG vs. AA | 0.52 (0.14-1.99) | 0.34 |  | 0% | 0.91 |
|  | Additive | 0.95 (0.81-1.13) | 0.59 |  | 0% | 0.56 |  |  | Additive | 1.11 (0.92-1.33) | 0.26 |  | 0% | 0.86 |
| rs4986791 | Dominant | 0.80 (0.61-1.05) | 0.11 |  | 0% | 0.88 |  | rs7045953 | Dominant | 0.98 (0.87-1.11) | 0.76 |  | 0% | 0.68 |
|  | Recessive | 2.21 (0.27-17.98) | 0.46 |  | 0% | 0.80 |  |  | Recessive | 0.78 (0.54-1.13) | 0.18 |  | 0% | 0.88 |
|  | CT vs. CC | 0.78 (0.59-1.04) | 0.09 |  | 0% | 0.87 |  |  | GA vs. AA | 1.00 (0.88-1.14) | 0.97 |  | 0% | 0.71 |
|  | TT vs. CC | 2.15 (0.27-17.55) | 0.47 |  | 0% | 0.81 |  |  | GG vs. AA | 0.78 (0.54-1.13) | 0.19 |  | 0% | 0.85 |
|  | Additive | 0.82 (0.62-1.08) | 0.15 |  | 0% | 0.68 |  |  | Additive | 0.96 (0.87-1.07) | 0.50 |  | 0% | 0.82 |

SNPs that were evaluated by three studies were shown here. Abbreviation: OR, odds ratio; CI, confidence interval; PCa, prostate cancer
